# Screening for the Risk of OSA in Periodontitis Patients. A Pilot Study

**DOI:** 10.3290/j.ohpd.b3125665

**Published:** 2022-06-13

**Authors:** Alexander R.E. Verhelst, Madeline X.F. Kosho, Ghizlane Aarab, Bruno G. Loos

**Affiliations:** a Periodontist, Department of Periodontology, Academic Centre for Dentistry Amsterdam (ACTA), University of Amsterdam and Vrije Universiteit, Amsterdam, The Netherlands. Conceived and designed the study, collected, analysed and interpreted the data, drafted the manuscript, critically revised the manuscript.; b PhD Student, Department of Periodontology, Academic Centre for Dentistry Amsterdam (ACTA), University of Amsterdam and Vrije Universiteit, Amsterdam, The Netherlands. Conceived and designed the study, collected, analysed and interpreted the data, drafted the manuscript, critically revised the manuscript.; c Professsor, Department of Orofacial Pain and Dysfunction, Academic Centre for Dentistry Amsterdam (ACTA), University of Amsterdam and Vrije Universiteit Amsterdam. Amsterdam, The Netherlands. Conceived and designed the study, critically revised the manuscript.; d Professsor, Department of Periodontology, Academic Centre for Dentistry Amsterdam (ACTA), University of Amsterdam and Vrije Universiteit Amsterdam. Amsterdam, The Netherlands. Conceived and designed the study, analysed and interpreted the data, drafted the manuscript, critically revised the manuscript.

**Keywords:** immune disorders, obstructive sleep apnea, obstructive sleep apnea syndrome, periodontitis

## Abstract

**Purpose:**

To determine the possibility of screening for the risk for Obstructive Sleep Apnea (OSA) in periodontitis patients.

**Materials and Methods:**

Periodontitis patients and non-periodontitis controls were recruited and asked to complete a validated screening questionnaire to calculate individual probabilities (%) of OSA. Also, for both groups, the risk for OSA was classified as low, medium and high.

**Results:**

Seventy periodontitis patients (49% male) and 77 controls (60% male) were included and both had an average age of 54 years. There was no statistically significant difference in the probability of the risk of OSA between periodontitis patients and controls, 38.6% ± 29.7%, and 34.2% ± 23.3%, respectively (p = 0.31). After sub-grouping individuals in “not high risk” (low plus intermediate) and “high OSA risk” categories, we observed statistically significantly more periodontitis patients than controls in the “high risk” category for OSA (21% vs 9%, p = 0.041, OR 2.73 [95% CI = 1.04 – 7.15]).

**Conclusion:**

These findings suggest that screening for OSA among periodontitis patients may help in early recognition of a “high risk” of OSA, but further research is needed.

Periodontitis is a complex chronic inflammatory disease, also considered a chronic immune disorder (CID), which results in the loss of tooth-supporting structures and the resorption of alveolar bone.^28,31^ Factors known to contribute to the risk for developing periodontitis are: dysbiotic biofilms, genetic and epigenetic variants, systemic diseases such as diabetes mellitus, and lifestyle-related factors, including obesity and smoking.^4,25,26^ Moreover, periodontitis patients are more prone to suffer from diabetes mellitus^34^ and atherosclerotic cardiovascular disease.^33^ Periodontitis is a complex disease, i.e. all of the risk factors described above may simultaneously interact with each other and determine immune fitness in individual patients.^25,26^

Obstructive sleep apnea (OSA) is defined as a recurrent obstruction of the upper airways, often resulting in oxygen desaturation and arousal (awakening) from sleep.^2^ OSA patients can suffer from a range of consequences of their condition, including not only complaints of snoring and excessive daytime sleepiness, but also symptoms of neurocognitive impairment and mood disturbance.^23^ Individuals with untreated OSA are at increased risk of several systemic diseases, such as heart failure,^17,18,29^ DM,^22^ and hypertension.^9^ In addition, individuals with OSA may suffer from other fatigue-related issues, such as car accidents.^15^

The golden standard for the diagnosis of OSA is polysomnography (PSG), which requires physiologic measurements of brain activity during sleep and measurements of the amount of airflow reductions and oxygen desaturation during sleep.^12^ Based on the PSG, an apnea-hypopnea index (AHI) is obtained and those with an AHI of at least 5 events/hour are diagnosed with OSA.^2^ Nevertheless, the symptoms and/or disease-related knowledge of the individual, of his/her family members or even of the family physician, are not always present.^39^ Consequently, the pathology of OSA and its diagnosis often goes undetected. Therefore, the prevalence of OSA is not clearly defined. Nevertheless, in a systematic review, the prevalence of OSA was estimated to be 49.0% for an AHI score of ≥ 5 events per hour and 28.5% for an AHI score of ≥ 15 events per hour among the Dutch population in the age range of 30 – 69 years.^5^

Several risk factors have been suggested for OSA, obesity being the strongest. An increase in body weight of 10% leads to a 6-times higher risk of having an AHI value of ≥ 15/h and might be associated with an increase of 32% in AHI score.^30^ Moreover, a reduction of 10% in body weight might yield a reduction of AHI of 26%.^19^ A recent review described that OSA patients show an increased prevalence of diabetes (15% in OSA patients vs 3% in a general population). Since reduced sleep can increase insulin resistance and/or glucose intolerance, it could have a direct relationship with OSA, but a causal link has not yet been established.^8^ When comparing sexes, two studies found males to be at an increased risk for high AHI values.^19,38^ Also, age is associated to OSA: people show an increased risk for having a higher AHI-value up to the 7th decade of life.^19,39^ In a systematic review assessing the influence of smoking on sleep and OSA, it was stated that smokers have a greater probability of OSA than non-smokers and former smokers.^10^ However, the latter paper also mentioned that the literature lacks strong evidence for the relation between smoking and OSA. Since OSA shares several of the above mentioned risk factors with periodontitis and co-morbidities, all related to a reduced immune function, it is conceivable that patients suffering from periodontitis are more prone to a higher risk for OSA than individuals without periodontitis.

Several studies have reported on the association between OSA and periodontitis. However, there are no studies investigating the prevalence of OSA or the risk of having OSA among periodontitis patients using the validated questionnaire developed by Eijsvogel et al.^13^ One article^27^ is available on the risk for OSA and periodontitis; however, it employed the less accurate STOP-BANG questionnaire. A moderate positive association between periodontitis and OSA was found in that study.

Therefore, the aim of this pilot study was to determine whether it is possible to screen periodontitis patients for the risk of having OSA, carried out at a periodontal clinic in a population predisposed to potentially suffer from OSA. To assess the risk for OSA, we used a validated risk-screening questionnaire which was originally validated against polysomnography (PSG); the purpose was not to diagnose OSA. Since periodontitis and OSA share risk factors, we hypothesized that a person’s risk for OSA would be higher if he or she has periodontitis.

## Material and Methods

### Patient Recruitment

For the current cross-sectional pilot study, patients were recruited over a period of 10 months at the dental clinics of the Academic Centre for Dentistry Amsterdam (ACTA) (March 2018 – January 2019). The study is a part of a larger study which investigates systemic conditions among subjects with and without periodontitis (study in progress). All participants received verbal and written information about the purpose of the study and provided informed consent. Participants had to be able to read and understand the Dutch language. The parent study was registered at the ClinicalTrials.gov (Identifier NCT03459638) and was approved by the Medical Ethics Committee (METC) of the VU University Medical Center (2017.490 (A2019.151) – NL62337.029.17). For this study, the STROBE guidelines were followed.^37^

All subjects ≥ 40 years of age, diagnosed with periodontitis and referred for treatment of periodontitis to the Department of Periodontology at ACTA, were asked to volunteer for this study. Patients were initially diagnosed with periodontitis if they fulfilled the criteria of the Centers for Disease Control and Prevention – American Academy of Periodontology (CDC-AAP) case definition.^14^ Based on the clinical measurements and the radiographic analysis, subjects that were diagnosed with periodontitis (≥ 2 interproximal sites with CAL ≥ 3 mm and ≥ 2 interproximal sites with PPD ≥ 4 mm [not on same tooth] or one site with PPD ≥ 5 mm) were asked to participate. Staging and grading was applied for each included periodontitis case according to criteria of the consensus report of the World Workshop on the Classification of Periodontal and Peri-Implant Diseases and Conditions.^28^

Control subjects were recruited consecutively among dental patients without periodontitis from the ACTA Clinics for General Dentistry, where appointments were scheduled for regular dental check-ups or restorative procedures. No efforts were made to match patients with control subjects with respect to age, sex or other characteristics. Control subjects, ≥ 40 years of age, were included if they did not fulfil any of the aforementioned criteria for case definition. In addition, these subjects showed no interproximal alveolar bone loss on ≤1-year-old dental bitewing radiographs.

### Clinical Procedures

Both periodontitis patients and control subjects underwent research-related examinations, which involved the following procedures and/or analyses:

- Measurement of systolic and diastolic blood pressure. Blood pressure was measured three times on the right arm with a digital sphygmomanometer (Omron; Kyoto, Japan). The blood pressure was defined as the average of the second and third measurement.
- Measurement of height and body weight, waist and neck circumference, and a questionnaire for demographic characteristics.
- Finger-prick for measuring HbA1c levels: a drop of blood was obtained with a finger prick (Greiner Bio-one Safety Lancet) and collected in a special collection tube (Hem-Col, Labonovum BV; Limmen, the Netherlands). The collection tube was sent to the laboratory for biochemical analysis by regular mail.

Recently, a new validated questionnaire consisting of 24 questions was introduced to screen for the risk of OSA. The questionnaire consists of items that best predict the risk of OSA.^13^ The risk of OSA was classified as low < 35%, intermediate 35–55%, and high >55%.^13^ Patients were provided with the OSA questionnaire after periodontal and clinical measurements were taken and the finger-prick procedure was performed (24 questions, supplement 1). All study subjects filled out the questionnaire privately. The questionnaire was labelled with a research number and took 10–15 min. The resulting risk for OSA was sent by letter to the patient, including a letter to their general practitioner if the OSA risk was high.

### Data Analysis

The primary outcome was the risk for OSA in periodontitis patients and controls. No sample calculation was performed, since no data were available about a possible relationship between periodontitis and OSA, using the validated OSA risk questionnaire. Therefore, this study should be considered a pilot study.

First, the risk of OSA was determined for each individual with the validated questionnaire, which also takes into account demographic data such as sex, age, weight, and neck circumference. Participants were assigned to one of the three pre-determined risk categories for OSA: low, intermediate or high.^13^ Subsequently, the high-risk category was assessed for its distribution in periodontitis patients and controls.

Data were analysed with SPSS 25.9.6.0.0 (IBM SPSS; Armonk, NY, USA). The means, standard deviations and frequency distributions were calculated. Background and OSA-related characteristics within the study population were compared with parametric and non-parametric tests (independent samples t-test and chi-squared test, respectively). Odds ratios (OR) with corresponding 95% confidence intervals (CI) were calculated when applicable. To further explore possible confounding effects of variables that were not included in the questionnaire, we performed a logistic regression analysis, creating three models. Model one corrected for smoking, the second model corrected for elevated HbA1c, and the third model corrected for both smoking and elevated HbA1c. We applied a cut-off value of HbA1c ≥ 7% (53 mmol/l), corresponding to the probability of having diabetes in the Dutch population, and avoiding the inclusion of possible false positive subjects.^36^

For all analyses, the significance level was set at p < 0.05.

## Results

### Background characteristics

**Fig 1:**
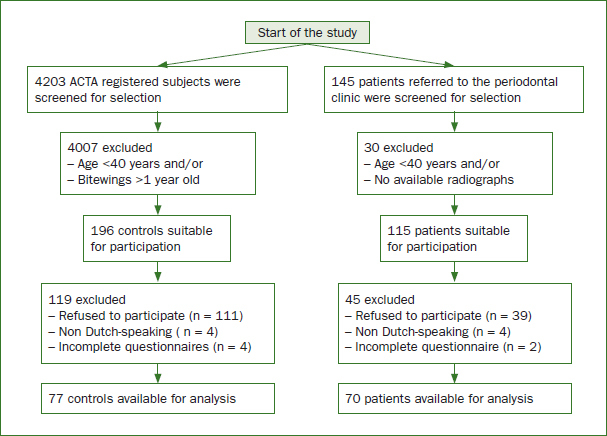
Flowchart of selection of periodontitis patients and controls.

In this study, 145 referred periodontitis patients were screened for suitability. Seventy cases fulfilled the inclusion criteria in the study period and were available for analysis. There were 4203 potential non-periodontitis controls screened for selection, of which 77 fulfilled all inclusion criteria and were available for analysis. Figure 1 presents a flowchart with details on patient recruitment and reasons for exclusion.

Background characteristics are presented in Table 1. The mean ages for controls and periodontitis patients were 53.6 and 54.3 years, respectively (p = 0.620). Overall differences between both groups in sex distribution, origin (European vs non-European) and education (primary, secondary and > secondary) were not statistically significant (p = 0.174, p = 0.678 and p = 0.105, respectively). The mean BMI was 26.0 kg/m^2^ for controls and 26.8 kg/m^2^ for periodontitis cases, and 39 controls (50.6%) and 40 periodontitis cases (57.1%) had a BMI of ≥ 25 kg/m^2^ (p = 0.277 and p = 0.430, respectively). In addition, when comparing cases and controls, no statistically significant difference regarding the prevalence of hypertension was found (p = 0.907). Among the periodontitis patients, there were statistically significantly more current smokers (34.3%), while this was 11.7% among controls (p = 0.001). A statistically significant higher proportion of periodontitis patients was found with elevated HbA1c compared to controls (27.1% vs 13%, p = 0.031).

**Table 1:** Background characteristics of the study population

	Control (n = 77)	Periodontitis (n = 70)	p-value
Age (years)	53.6 ± 9.3	54.3 ± 8.5	0.620
**Sex**			0.174
Male	46 (59.7)	34 (48.6)	
Female	31 (40.3)	36 (51.4)	
**Origin**			0.678
European	52 (67.5)	45 (64.3)	
Non-European	25 (32.5)	25 (35.7)	
**Education** ^a^			0.105
Primary	9 (11.7)	16 (22.9)	
Secondary	21 (27.3)	22 (31.4)	
>Secondary	47 (61.0)	32 (45.7)	
**BMI (kg/m^2^)**	26.0 ± 4.8	26.8 ± 4.3	0.277
BMI ≥ 25 kg/m^2^	39 (50.6)	40 (57.1)	0.430
**Smoking status**			0.001
Current	9 (11.7)	24 (34.3)	
Former	25 (32.5)	25 (35.7)	
Never	43 (55.8)	21 (30.0)	
**Hypertension** *	26 (33.8)	23 (32.9)	0.907
**HbA1c** ≥ **7**%**	10 (13.0)	19 (27.1)	0.031

Values represent means ± SD or numbers (%) of subjects. BMI: body mass index. *Based on clinical measurements: ≥ 140 mmHg systolic and/or ≥ 90 mm Hg diastolic (average of 2nd and 3rd measurement). **HbA1c level of ≥ 7% is equal to ≥ 53 mmol/l. ^a^Primary: primary education or preparatory secondary vocational education. Secondary: higher secondary general education, pre-university education. >Secondary: beyond secondary education.

The periodontal condition is presented in Table 2. Periodontitis patients had statistically significantly fewer teeth than controls (26.3 vs 27.7, p < 0.001), on average 2.7 teeth with ≥ 50% bone loss, 7.6 teeth with PPD ≥ 6 mm and 15.5 sites with PPD ≥ 6 mm. Controls had no alveolar bone loss, nor any site with PPD of ≥ 6 mm.

**Table 2:** Clinical periodontal characteristics of periodontitis patients and controls

	Control (n = 77)	Periodontitis (n = 70)	p-value
# Teeth	27.7 ± 2.2	26.3 ± 2.6	<0.001
# Teeth with ≥ 50% bone loss	NA	2.7 ± 3.4	NA
# Teeth with PPD ≥ 6 mm	NA	7.6 ± 6.0	NA
# Sites with PPD ≥ 6 mm	NA	15.5 ± 15.8	NA
			
Stage III Stage IV	NA NA	57 (81.4) 13 (18.6)	NA NA
			
Grade B	NA	24 (34.4)	NA
Grade C	NA	46 (65.7)	NA

Values represent means ± standard deviation or number (%) of patients. NA: not applicable; PPD: probing pocket depth.

Of the 70 periodontitis patients, 81.4% were classified as stage III and 18.6% were stage IV. Moreover, 34.4% were grade B and 65.7% were grade C.

### Risk for OSA

The mean risk for OSA, calculated via the validated questionnaire is presented in Table 3. The mean risk for OSA was 34.2% for controls and 38.6% for periodontitis patients. This difference was not statistically significant (p = 0.307). When assigning subjects to predetermined risk categories, a trend could be observed, where almost the same proportion of subjects belonged to the low-risk category (48.1% for periodontitis patients and 47.1% for controls). A higher proportion of controls (42.9%) than periodontitis cases (31.4%) proved to have an intermediate risk of OSA, while more periodontitis patients than controls were found in the high OSA risk group (21.4% and 9.1%, respectively). However, differences in proportions were not statistically significant (p = 0.081).

**Table 3:** Risk for OSA, determined with the validated OSA risk questionnaire^13^ and frequency distributions of subjects in risk categories

	Controls (n = 77)	Periodontitis (n = 70)	OR (95% CI)	p-value
Risk for OSA (%)	34.2 ± 23.3	38.6 ± 29.7		0.307
Risk category for OSA				
Low	37 (48.1)	33 (47.1)		
Intermediate	33 (42.9)	22 (31.4)		0.081
High	7 (9.1)	15 (21.4)		
Risk for OSA				
Not high	70 (90.1)	55 (78.6)		
High^1^	7 (9.1)	15 (21.4)	2.73 (1.04 – 7.15)	0.041

Values represent means ± SD or numbers (%) of subjects. CI: confidence interval; OR: odds ratio; OSA: obstructive sleep apnea. ^1^Model 1: adjusted for smoking: OR (95% CI) = 2.72 (1.01 – 7.35); p = 0.049. Model 2: adjusted for elevated HbA1c: OR (95% CI) = 2.24 (0.82 – 6.07); p = 0.114. Model 3: adjusted for smoking and elevated HbA1c: OR (95% CI) = 2.08 (0.74 – 5.82); p = 0.165.

When combining the low and intermediate OSA risk groups to a ‘not high risk’ group, a statistically significantly higher percentage for a high risk of OSA was observed for periodontitis patients than for controls (21.4% and 9.1%) with an OR of 2.73 (95% CI = 1.04–7.15, p = 0.041). After adjusting for smoking, the frequency of periodontitis patients in the high OSA risk category remained statistically significantly increased, compared to the frequency of control subjects (OR_adj_ = 2.72, 95% CI_adj_ = 1.01–7.35, p_adj_ = 0.049). However, when adjusting for elevated HbA1c, the distribution of periodontitis patients and control subjects in the high OSA risk category lost statistical significance (OR_adj_ = 2.24, 95% CI_adj_ = 0.82–6.07, p_adj_ = 0.114). Again, when we adjusted for both smoking and elevated HbA1c, the frequencies of periodontitis patients and non-periodontitis subjects in the high risk category were not statistically significantly different (OR_adj_ = 2.08, 95% CI_adj_ 0.74–5.82, p_adj_ = 0.165).

## Discussion

In this pilot study, the risk of OSA was assessed in periodontitis patients and in non-periodontitis controls. Based on the outcomes of the validated questionnaire employed, the frequency of a high risk for OSA was statistically significantly higher in periodontitis patients than among controls. To date, only limited research on a possible association between OSA and periodontitis has been done. So far, several studies have described the prevalence of periodontitis in an already diagnosed OSA population, whereas the reverse – the prevalence of OSA in periodontitis patients – has not yet been investigated.^7^ Some studies found a (weak) association between periodontitis and OSA,^1,16,34^ while one study could not confirm this suggested relationship.^24^ Nevertheless, in a meta-analysis of four studies, a statistically significant association between OSA and periodontitis was revealed, with an OR of 1.65 (95% CI 1.11 – 2.46) for OSA in periodontitis patients,^3^ although it was stated that due to extensive heterogeneity more studies are needed. We are the first to report that severe periodontitis patients (stage ≥ III) may have a high risk of OSA.

One of the limitations of the study is that the main finding – periodontitis patients more often had a “high risk” for OSA – is based on 15 out of 70 periodontitis patients vs 7 out of 77 controls. Therefore, the current study must be considered a pilot study and further confirmational research is needed. Secondly, our study did not aim to diagnose OSA. Polysomnography is the golden standard for diagnosing OSA. Nevertheless cheaper and less time-consuming alternatives, such as validated questionnaires, can be of added value in pre-selecting potential cases or for excluding those without OSA. Thus, the current questionnaire was developed as a screening tool and risk estimator.^13^ The questionnaire was validated in a Dutch population of healthy blue- and white-collar workers. For the current study’s investigation of a possible association between periodontitis and OSA, we only used the questionnaire to assess the risk for OSA. With an overall specificity of 90.1% and 95.5% for the high OSA risk group,^13^ this questionnaire is very well applicable in a clinical dental context. Nevertheless, another limitation may be that all patients and controls were recruited from a university setting and the generalisability is currently unknown.

In the current study, smoking was not a statistically significant potential confounder associated with high risk of OSA in periodontitis patients. Earlier studies noted that it was still unclear whether smoking per se is a risk factor for OSA.^10,20^ On the other hand, elevated HbA1c was a statistically significant confounding factor in the finding of high risk of OSA in periodontitis patients. The association between OSA and diabetes has been found before.^32^ Interestingly, having OSA may lead to a higher prevalence of diabetes. However, the underlying mechanisms are poorly understood and the relationship is suggested to be bi-directional.^8^

A recent narrative review linked reduced immune function with sleep disorders.^6^ Despite the fact that most of the possible mechanisms remain (partly) unclear, it was stated that the immune system and sleep are bi-directionally related and influenced by each other. Moreover, a number of medications for the treatment of various diseases may have sleep-disturbing effects, thereby potentially decreasing the immune-supportive effects of sleep.^6^ In a study conducted among 574 university students to investigate their perceived immune status, it appeared that sleep apnea was significantly associated with a weakened immune system.^11^

Due to the fact that several co-morbidities, including periodontitis, are associated with OSA, it can have a major impact on the individual’s life in terms of health as well as socioeconomic aspects.^21^ It is clear that adequate treatment may reduce the consequences of sleep apnea. However, if socially and economically significant reductions in morbidity, mortality and social impact are to be achieved, early disease identification and management are needed. Therefore, evaluation of a general screening and disease-management programme is a prerequisite.^21^ From this perspective, screening by means of a questionnaire among periodontitis patients in a dental practice or in a periodontology referral practice, could be useful for early detection and, as a consequence, result in earlier diagnosis and treatment, with less co-morbidities, less socioeconomic impact and an improved quality of life for patients.

## Conclusion

In the current pilot study, periodontitis patients were found to more often have a high risk for OSA than non-periodontitis controls. Within the limitations of the current study, our findings suggest that screening for the risk of OSA among periodontitis patients can be of added value to prevent major co-morbidities associated with OSA and thereby contribute to general health.

## Supplement 1: Validated questionnaire on the risk of osa, as used in the current study^13^

This questionnaire contains questions about your health, your sleep and your well-being

It’s important to answer all questions, even if they seem similar. All answers are needed to calculate your risk for sleep apnea!

The questionnaire starts with some general questions about you and your health.

What is your age?

What gender are you?

What is your weight?

What is your height?

What is the circumference of your neck in cm?

Do you have an increased blood pressure?

Yes

No

Don’t know

### Questions 3–8 are about your well-being, your functioning and possible fatigue. Please circle the best fitting answer.

How often do you get up, feeling you are tired and have not had enough sleep?

Almost every day

3-4 times a week

1-2 times a week

1-2 times a month

Never or very rarely

When you are awake, do you often feel tired, not rested or not alert?

Almost every day

3-4 times a week

1-2 times a week

1-2 times a month

Never or very rarely

Do you often feel tired, not rested or sleepy during the day?

Yes

No

*Sense of well-being during the day*

Normal

Slightly decreased

Markedly decreased

Very decreased

*Functioning (physical and mental) during the day*

Normal

Slightly decreased

Markedly decreased

Very decreased

*Sleepiness during the day*

Mild

Considerable

Intense

### Questions 9–14 are about your sleep and about snoring.

Do you snore?

Yes

No

Don’t know

When you snore, is this:

Slightly louder than breathing?

The same volume as talking?

Louder than talking?

Very loud – can be heard in adjacent rooms?

Do you snore loudly (louder than talking and loud enough to be heard through closed doors)?

Yes

No

How often do you snore?

Almost every day

3-4 times a week

1-2 times a week

1-2 times a month

Never or very rarely

Has your snoring ever disturbed other people?

Yes

No

Don’t know

Has anyone ever seen you stop breathing when you were asleep?

Yes

No

These questions are about how you experience possible sleeping issues. Please indicate at the following situations how problematic the situation is for you (assuming you experience this at least 3 times per week).

Sleep induction (time it takes you to fall asleep after turning off the lights)

No problem

Slightly delayed

Markedly delayed

Very delayed or did not sleep at all

Awakening during the night

No problem

Minor problem

Considerable problem

Serious problem or did not sleep at all

Final awakening earlier than desired

Not earlier

A little earlier

Markedly earlier

Much earlier or did not sleep at all

Total sleep duration

Sufficient

Slightly insufficient

Markedly insufficient

Very insufficient or did not sleep at all

Overall quality of sleep (no matter how long you slept)

Satisfactory

Slightly unsatisfactory

Markedly unsatisfactory

Very unsatisfactory or did not sleep at all
